# Controlled attenuation parameter value-based diagnostic algorithm improves the accuracy of liver stiffness measurement in chronic hepatitis B patients

**DOI:** 10.18632/aging.103522

**Published:** 2020-08-24

**Authors:** Yaoxin Fan, Lin Wang, Yang Ding, Qiuju Sheng, Chong Zhang, Yanwei Li, Chao Han, Xiaoguang Dou

**Affiliations:** 1 Department of Infectious Diseases, Shengjing Hospital of China Medical University, Shenyang Liaoning Province, China; 2 Department of Health Management, Shengjing Hospital of China Medical University, Shenyang Liaoning Province, China

**Keywords:** diagnostic algorithm, liver fibrosis measurement, chronic hepatitis B, controlled attenuation parameter

## Abstract

Liver stiffness measurement (LSM) frequently overestimates the severity of liver fibrosis because of steatosis. However, the impact of the controlled attenuation parameter (CAP) on liver stiffness cutoff values remains unknown; CAP was used to quantify and diagnose the severity of hepatic steatosis. The study was conducted to determine the effect of CAP on liver stiffness cutoff values in chronic hepatitis B (CHB) patients. A retrospective cross-sectional study was performed in liver biopsy-proven CHB patients. The median LSM (kPa) in the elevated CAP group was higher than that in the normal CAP group at the same fibrosis stage. For S2-4, the area under the receiver operating characteristic (AUROC) curve of LSM was 0.78 and 0.72 in the normal and elevated CAP groups, respectively. When a cutoff value of 8.9 kPa was used, the diagnostic accuracy was 77.82% and 63.41% in the normal and elevated CAP groups, respectively. Compared with the alanine transaminase (ALT)-based LSM algorithm, the CAP-based LSM algorithm had a similar correct diagnosis rate (33.64% vs. 33.94%, respectively) but a lower misdiagnosis rate (16.97% vs. 20.30%, respectively). The new CAP-based LSM diagnostic algorithm will improve the diagnostic accuracy of liver fibrosis in CHB patients.

## INTRODUCTION

Chronic hepatitis B (CHB) is a major cause of liver cirrhosis and hepatocellular carcinoma (HCC) worldwide. CHB accounts for 66% of deaths from chronic hepatitis [1, 2]. The correct diagnosis of liver fibrosis and HCC is one of the most important steps in the management and treatment of CHB. Liver stiffness measurement (LSM) by transient elastography (TE) is a noninvasive first-line tool to assess liver fibrosis [3, 4]. LSM can be performed quickly and easily in outpatients with chronic hepatitis B infection [5]. However, LSM diagnostic outcomes could be strongly influenced by many factors, including inflammation [6] and steatosis. Therefore, alanine transaminase (ALT) levels must be considered when interpreting the cutoff values for LSM [7]. Different LSM cutoff values based on different ALT levels have been developed for the assessment of liver fibrosis in CHB patents and are recommended by the European Association for the Study of the Liver (EASL) [3].

However, the ALT-based LSM algorithm has several limitations in its application. For example, LSM values may be inaccurate due to large fluctuations during acute hepatitis, ALT flares and the use of liver-protecting drugs [8]. Therefore, we suggest that other significant factors should be considered when interpreting LSM results.

In addition to liver inflammation, steatosis and liver fibrosis are factors that affect LSM values. Controlled attenuation parameter (CAP) is an ultrasound-based quantitative measurement used in conjunction with LSM [9]. CAP is a reliable value used to quantify and diagnose the severity of hepatic steatosis [10, 11]. A higher CAP value is associated with a higher degree of hepatic steatosis. Importantly, a considerable number of CHB patients have metabolic syndrome and hepatic steatosis [12, 13]. A recent study illustrated that in CHB patients with moderate to severe liver steatosis, LSM value was overestimated and affected the diagnosis of significant fibrosis when CAP value was not less than 268 dB/m [14]. However, the effect of CAP on LSM cutoff values is still unknown. In this study, we sought to explore the effect of CAP on LSM cutoff values in CHB patients and to develop an algorithm to improve the diagnostic accuracy of LSM.

## RESULTS

### Patient characteristics

**Figure 1:**
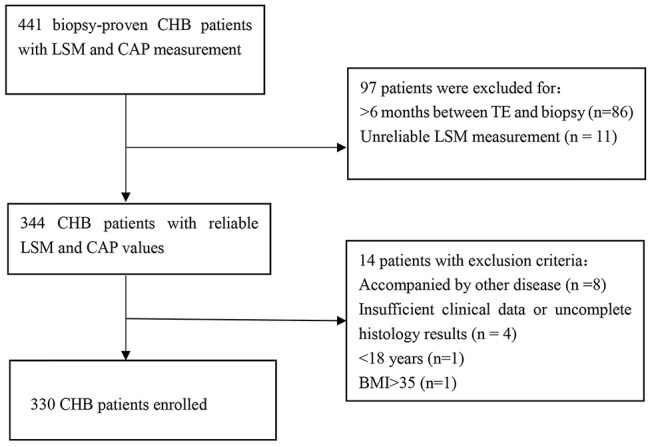
**Flowchart of the study population selection.**

A total of 441 CHB patients who had a liver biopsy and LSM for primary liver disease were recruited for this study, and 330 CHB patients were ultimately enrolled in the study (Figure 1).

The mean age was 36 years, with a predominance of males (61.21%). Overall, 41.52% of the patients had significant liver necroinflammation (G2-4), and 31.21% of the patients had significant fibrosis (S2-4). The baseline characteristics of the patients are shown in Table 1.

**Table 1:** Patient characteristics of chronic hepatitis B (n=330).

**Variables**	**Values**
Age (years)	36.0 (30.0, 44.0)
Sex (male%)	202 (61.2%)
ALT (IU/L)	37.0 (26.0, 64.3)
AST (U/L)	28.0 (22.0, 39.0)
GGT (U/L)	22.0 (16.0, 37.0)
ALP (U/L)	75.9 (63.3, 95.4)
TB(μ/L)	13.0 (9.8, 16.7)
Total cholesterol (mmol/L)	4.3 (3.8, 4.9)
BMI (kg/m^2^)	23.7±3.4
Liver stiffness (kPa)	7.4 (5.7, 9.7)
CAP (dB/m)	224.1 (206.6, 247.9)
Grade of necroinflammation	
G0	0 (0%)
G1	193 (58.5%)
G2	105 (31.8%)
G3	32 (9.7%)
G4	0 (0.0%)
Stage of fibrosis	
S0	118 (35.8%)
S1	109 (33.0%)
S2	59 (17.9%)
S3	27 (8.2%)
S4	17 (5.2%)

Data are expressed as the means ± standard deviation or the medians (interquartile range, IQR). ALT: alanine aminotransferase; AST: aspartate aminotransferase; GGT: gamma-glutamyl transferase; ALP, alkaline phosphatase; TB, total bilirubin; S: stage of fibrosis; BMI, body mass index; CAP, controlled attenuation parameter

### Factors associated with CAP

CAP was significantly correlated with sex (r=-0.216, P <0.001), ALT (r=0.232, P<0.001), aspartate aminotransferase (AST) (r=0.151, P=0.006), glutamyl transpeptidase (GGT) (r=0.384, P<0.001), alkaline phosphatase (ALP) (r=0.207,P<0.001), total cholesterol(TC) (r=0.125, P=0.024), liver fibrosis (r=0.119, P=0.03), body mass index (BMI) (r=0.663, P <0.001) and LSM (r=0.279, P <0.001), while there was no correlation with age, total bilirubin(TB) or liver necroinflammation (P>0.05 for all). Based on the univariate linear regression analysis, sex (male), ALT, GGT, ALP, BMI and LSM were all correlated with CAP (P<0.05). However, only BMI was independently significantly correlated with CAP (P<0.01) (Table 2) by multivariate linear regression analysis.

**Table 2:** Univariate and multivariate linear regression of risk factors associated with controlled attenuated parameter.

**Variables**	**Univariate analysis**			**Multivariate analysis**		
	**β**	**Standard error**	**P-value**	**β**	**Standard error**	**P-value**
Age (years)	0.048	0.186	0.389			
Sex	-0.213	3.705	<0.001	-0.021	3.029	0.635
ALT (IU/L)	0.183	0.055	0.001	0.018	0.049	0.710
GGT (U/L)	0.281	0.052	<0.001	0.076	0.049	0.131
ALP (U/L)	0.172	0.072	0.002	0.036	0.058	0.409
TB (μ/L)	-0.004	0.297	0.937			
TC (mmol/L)	0.047	1.701	0.397			
BMI (kg/m ^2^)	0.663	0.407	<0.001	0.611	0.431	<0.001
Liver stiffness (kPa)	0.280	0.346	<0.001	0.077	0.297	0.091

ALT: alanine aminotransferase; GGT: gamma-glutamyl transferase; ALP, alkaline phosphatase; TB, total bilirubin; BMI, body mass index; TC, total cholesterol

### Factors associated with LSM

The median LSM values were 6.63 (5.25-8.22) for S0, 6.67 (5.28-8.93) for S1, 9.32 (7.08-12.38) for S2, 10.66 (8.14-17.8) for S3 and 16.45 (7.88-21.24) for S4. LSM was significantly correlated with ALT (r=0.329, P<0.001), AST (r=0.292, P<0.001), GGT (r=0.358, P<0.001), ALP (r=0.161, P=0.003), TB (r=0.101, P=0.046), liver necroinflammation (r=0.337, P<0.001), liver fibrosis (r=0.435, P<0.001), CAP (r=0.279, P<0.001) and BMI (r=0.264, P<0.001); there was no correlation with age, sex or total cholesterol (P>0.05 for all).

Univariate and multivariate linear regression analyses were performed to identify factors correlated with LSM in the entire CHB cohort and in the fibrosis stage subgroups of S0-1 and S2-4 (Table 3). LSM values were significantly correlated with ALT (P<0.001) and GGT (P=0.007) in the entire cohort. ALT (P=0.004) and CAP (P=0.041) were significantly correlated with LSM values in the S0-1 subgroup but not in the S2-4 subgroup.

**Table 3:** Univariate and multivariate linear regression of risk factors associated with liver stiffness measurement.

**Variables**	**Univariate analysis**			**Multivariate analysis**		
	**β**	**Standard error**	**P-value**	**β**	**Standard error**	**P-value**
**Entire CHB Cohort (n=330)**						
Age (years)	-0.124	0.577	0.024	0.070	0.026	0.169
Sex	0.058	0.028	0.295			
ALT (IU/L)	0.353	0.008	<0.001	0.241	0.009	<0.001
GGT (U/L)	0.346	0.008	<0.001	0.161	0.009	0.007
ALP (U/L)	0.171	0.011	0.002	0.041	0.011	0.430
TB (μ/L)	0.088	0.045	0.112			
TC(mmol/L)	-0.009	0.261	0.875			
BMI (kg/m^2)^	0.264	0.081	<0.001	0.097	0.101	0.147
CAP (dB/m)	0.28	0.008	<0.001	0.116	0.010	0.085
**S0-1 CHB (n=227)**						
Age (years)	0.036	0.022	0.590			
Sex	0.005	0.426	0.937			
ALT (IU/L)	0.299	0.008	<0.001	0.209	0.008	0.004
GGT (U/L)	0.247	0.008	<0.001	0.059	0.009	0.417
ALP (U/L)	0.055	0.009	0.412			
TB (μ/L)	0.053	0.032	0.430			
TC (mmol/L)	0.064	0.188	0.337			
BMI (kg/m^2^)	0.274	0.060	<0.001	0.103	0.079	0.227
CAP (dB/m)	0.323	0.006	<0.001	0.179	0.008	0.041
**S2-4 CHB (n=103)**						
Age (years)	-0.003	0.066	0.979			
Sex	-0.229	1.391	0.020	-0.140	1.393	0.152
ALT (IU/L)	0.289	0.015	0.003	0.203	0.016	0.050
GGT (U/L)	0.288	0.014	0.003	0.119	0.016	0.267
ALP (U/L)	0.095	0.026	0.338			
TB (μ/L)	0.084	0.119	0.398			
TC (mmol/L)	-0.057	0.656	0.569			
BMI (kg/m^2^)	0.277	0.191	0.005	0.140	0.239	0.246
CAP (dB/m)	0.217	0.019	0.028	0.092	0.023	0.436

ALT: alanine aminotransferase; GGT: gamma-glutamyl transferase; ALP, alkaline phosphatase; TB, total bilirubin; BMI, body mass index; CAP, controlled attenuation parameter; TC, total cholesterol

### Effect of CAP on LSM values

**Figure 2:**
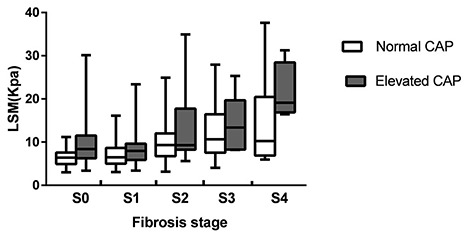
**LSM values in normal and elevated CAP groups according to fibrosis stage.**

Because CAP was associated with LSM, the effect of CAP on LSM values in the same stages of liver fibrosis was evaluated. Based on the CAP value, the patients were further classified into the normal CAP group (< 248 dB/m) and the elevated CAP group (≥ 248 dB/m). The median LSM values were 6.42 and 8.41 kPa (P<0.001) in CHB patients at stage S0; 6.50 kPa and 7.96 kPa (P=0.02) in CHB patients at stage S1; 9.32 kPa and 9.28 kPa (P=0.29) in CHB patients at stage S2; 10.66 kPa and 13.38 kPa (P=0.51) in CHB patients at stage S3; and 10.26 kPa and 19.11 kPa (P=0.20) in CHB patients at stage S4 (Figure 2). Higher LSM values were correlated with higher CAP values, especially in lower-stage patients (S0-1). This finding suggested that the LSM values could be overestimated in CHB patients with higher CAP values.

### Effect of CAP on LSM diagnostic performance

The study further evaluated the effect of CAP on LSM diagnostic performance. In the entire CHB cohort, the area under the receiver operating characteristic (AUROC) curve of LSM for the diagnosis of S2-4 was 0.77 (0.72-0.83). Using the Youden Index of transient elastography at 8.9 kPa, the sensitivity and specificity were 64.08% and 79.30%, respectively. For S3-4, the AUROC of LSM was 0.77 (0.69-0.85). Using the Youden Index of transient elastography at 10.1 kPa, the sensitivity and specificity were 61.36% and 82.87%, respectively (Table 4, upper).

**Table 4:** Different optimal cutoff values for different fibrosis stage in different CAP groups.

**Fibrosis stage**	**AUROC (95%CI)**	**Parameter**	**Cutoff (KPa)**	**Sensitivity (%)**	**Specificity (%)**	**PPV (%)**	**NPV (%)**	**Diagnostic accuracy (%)**	**LR (+)**	**LR (-)**
**All cases**										
		Sn	4.7	90.09	16.95	66.09	48.77	63.94	1.08	0.58
S1	0.65	Sp	10.1	30.66	90.68	85.53	42.13	52.12	3.29	0.76
	(0.60-0.71)	Youden Index	8.6	48.11	81.36	82.26	46.60	59.70	2.58	0.64
		Sn	5.8	90.29	33.04	37.96	88.24	50.91	1.35	0.29
S2	0.77	Sp	10.6	43.69	90.31	67.17	77.95	75.76	4.51	0.62
	(0.72-0.83)	Youden Index	8.9	64.08	79.30	58.41	82.95	74.55	3.09	0.45
		Sn	6.0	90.91	32.17	17.09	95.84	39.70	1.34	0.28
S3	0.77	Sp	12.1	50.00	90.21	43.99	92.15	84.85	5.11	0.55
	(0.69-0.85)	Youden Index	10.1	61.36	82.87	35.52	93.31	86.67	3.58	0.47
		Sn	6.0	94.12	29.71	6.78	98.94	33.03	1.34	0.20
S4	0.79	Sp	12.9	52.94	90.10	22.50	97.24	88.18	5.35	0.52
	(0.67-0.91)	Youden Index	10.1	70.59	79.55	15.78	98.03	79.09	3.45	0.37
**CAP<248**										
S1	0.66	Sn	4.5	90.32	15.05	63.93	48.26	62.10	1.06	0.64
	(0.59-0.73)	Sp	9.0	40.00	90.32	87.32	47.46	58.87	4.13	0.66
		Youden Index	8.6	43.23	89.25	87.02	48.54	60.48	4.02	0.64
S2	0.78	Sn	5.5	90.41	34.29	36.47	89.55	50.81	1.38	0.28
	(0.71-0.85)	Sp	9.5	53.42	90.29	69.65	82.29	79.44	5.50	0.52
		Youden Index	8.9	61.64	84.57	62.50	84.09	77.82	4.00	0.45
S3	0.77	Sn	5.9	91.18	35.51	18.34	96.20	43.15	1.41	0.25
	(0.68-0.87)	Sp	10.9	47.06	90.19	43.25	91.47	84.27	4.80	0.59
		Youden Index	10.1	58.82	87.38	42.55	93.03	83.47	4.66	0.47
S4	0.76	Sn	6.0	92.31	34.47	7.23	98.78	37.10	1.41	0.22
	(0.63-0.90)	Sp	12.1	46.15	90.21	20.68	96.80	87.90	4.72	0.60
		Youden Index	10.1	61.54	83.40	17.01	97.51	82.26	3.71	0.46
**CAP≥248**										
S1	0.58	Sn	5.7	91.23	20.00	72.22	50.01	69.51	1.14	0.44
	(0.45-0.71)	Sp	12.6	29.82	92.00	89.47	36.51	48.78	3.73	0.76
		Youden Index	14.8	26.32	96.00	93.75	36.37	30.49	6.58	0.77
S2	0.72	Sn	8.0	90.00	48.08	50.01	89.28	63.41	1.73	0.21
	(0.61-0.84)	Sp	12.6	46.67	90.38	73.68	74.60	74.39	4.85	0.59
		Youden Index	8.0	90.00	48.08	50.01	89.28	63.41	1.73	0.21
S3	0.80	Sn	8.3	90.00	43.06	18.01	96.87	48.78	1.58	0.23
	(0.66-0.94)	Sp	19.0	30.00	90.28	30.01	90.27	82.93	3.09	0.78
		Youden Index	16.4	70.00	88.89	46.68	95.52	86.59	6.30	0.34
S4	0.91	Sn	16.4	100.00	85.90	26.68	100.00	86.59	7.09	0.00
	(0.84-0.98)	Sp	20.0	50.00	91.03	22.24	97.26	89.02	5.57	0.55
		Youden Index	16.4	100.00	85.90	26.68	100.00	86.59	7.09	0.00

When considering the effect of CAP, the AUROC of LSM for the diagnosis of S2-4 was 0.78 (0.71-0.85) in the normal CAP group and 0.72 (0.61-0.84) in the elevated CAP group. When a cutoff value of 8.9 kPa was used, the sensitivity and specificity of LSM were 61.64% and 84.57% in the normal CAP group and 70% and 59.62% in the elevated CAP group, respectively. The diagnostic accuracy was 77.82% in the normal CAP group and 63.41% in the elevated CAP group. For the diagnosis of S3-4, the AUROC of LSM was 0.77 (0.68-0.87) in the normal CAP group and 0.80 (0.66-0.94) in the elevated CAP group. When a cutoff value of 10.1 kPa was used, the sensitivity and specificity of LSM were 58.82% and 87.38% in the normal CAP group and 70% and 69.44% in the elevated CAP group, respectively. The diagnostic accuracy was 83.47% in the normal CAP group and 69.51% in the elevated CAP group. In summary, patients with normal CAP values had a higher diagnostic accuracy than patients with elevated CAP values. CAP influenced the LSM diagnostic performance.

### Optimal cutoff values of LSM based on different CAP values

The Youden Index and at least 90% sensitivity and 90% specificity were used as the optimal cutoff values for LSM to evaluate the liver fibrosis stage [15]. The values for the sensitivity, specificity, positive predictive value (PPV), negative predictive value (NPV) and diagnostic accuracy (DA) are listed in Table 4. In the normal CAP group, for S≥S1, the cutoff values ranged from 4.5-9.0 kPa; for S≥S2, the cutoff values ranged from 5.5-9.5 kPa; for S≥S3, the cutoff values ranged from 5.9-10.9 kPa; and for S=S4, the cutoff values ranged from 6.0-12.1 kPa. In the elevated CAP group, for S≥S1, the cutoff values ranged from 5.7-14.8 kPa; for S≥S2, the cutoff values ranged from 8.0-12.6 kPa; for S≥S3, the cutoff values ranged from 8.3-19.0 kPa; and for S=S4, the cutoff values ranged from 16.4-20 kPa.

### Diagnostic flowchart using LSM accounting for CAP

**Figure 3:**
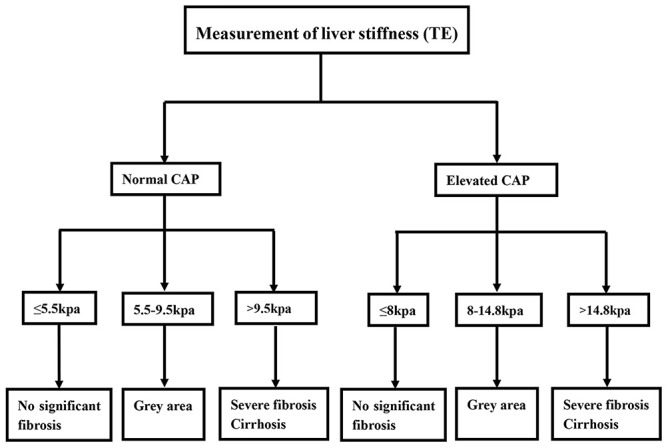
**Diagnostic flowchart for diagnosis and exclusion liver fibrosis in chronic hepatitis B virus infection based on LSM and CAP values.**

Based on the results of the above analysis, we developed a liver fibrosis diagnostic flowchart using the LSM and CAP. For patients with normal CAP values, an LSM of ≤5.5 kPa was defined as no significant fibrosis (no or mild fibrosis), an LSM of 5.5-9.5 kPa was defined as a gray area, and an LSM of > 9.5 kPa was defined as severe fibrosis or cirrhosis. For patients with elevated CAP values, an LSM of ≤8 kPa was defined as no or mild fibrosis, an LSM of 8.0-14.8 kPa was defined as a gray area, and an LSM of >14.8 kPa was defined as severe fibrosis or cirrhosis (Figure 3).

### Comparison of diagnostic performance using LSM, CAP or ALT level

The ALT-based LSM algorithm was recommended to diagnose or exclude severe liver fibrosis using LSM [5]. In the study, 179 patients had normal ALT levels, and 151 patients had elevated ALT levels. Based on the ALT-based LSM algorithm, in the normal ALT group, 64 patients had no or mild fibrosis (LSM<6 kPa), 74 patients had an uncertain diagnosis (6<LSM<9 kPa), and 41 patients had severe fibrosis or cirrhosis (LSM≥9 kPa) in the normal ALT group. In the elevated ALT group, 35 patients had no or mild fibrosis (LSM<6 kPa), 77 patients had an uncertain diagnosis (6<LSM<12 kPa), and 39 patients had severe fibrosis or cirrhosis (LSM≥12 kPa). In the normal ALT group, 68 (37.99%) patients had no significant fibrosis or a correct diagnosis of severe liver fibrosis/cirrhosis, 74 (41.34%) patients had an ambiguous diagnosis, and 37 (20.67%) patients were misdiagnosed. In the elevated ALT group, 44 (29.14%) patients had no significant fibrosis or a correct diagnosis of severe liver fibrosis/cirrhosis, 77 (50.99%) patients had an ambiguous diagnosis, and 30 (19.87%) patients were misdiagnosed.

**Figure 4:**
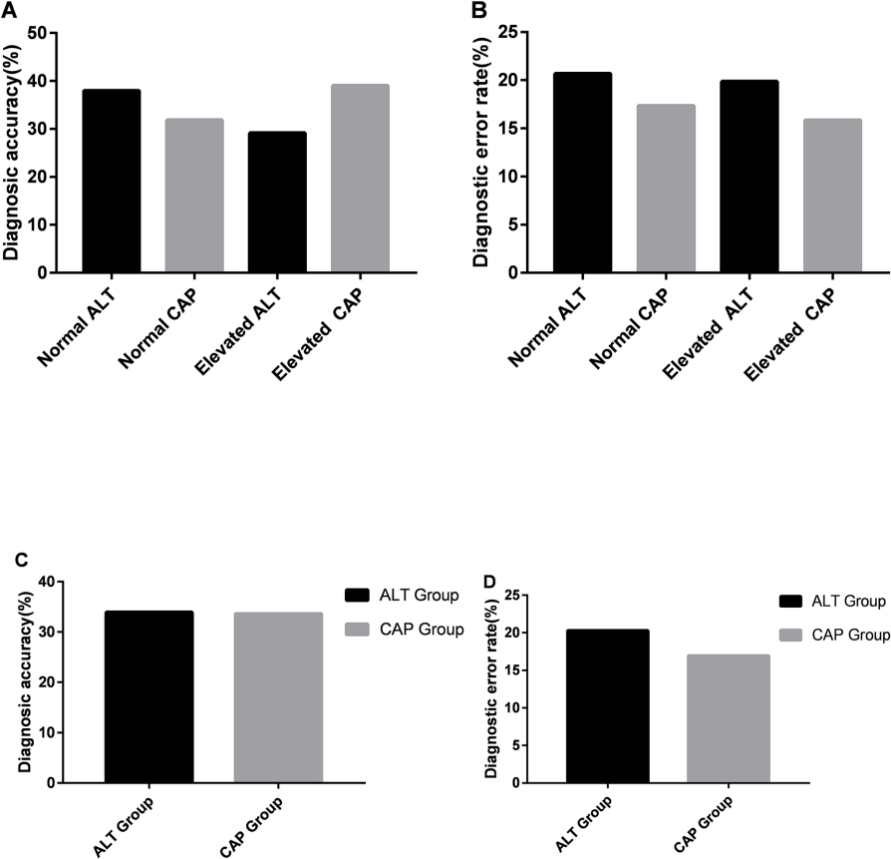
**The comparison of diagnostic performance between the ALT-based LSM algorithm and CAP-based LSM algorithm.** The comparison of diagnostic accuracy (**A**) and diagnosis error rate (**B**) in different subgroups. The comparison of diagnostic accuracy (**C**) and diagnostic error rate (**D**) of the ALT-based LSM algorithm and CAP-based LSM algorithm.

Based on the CAP-based LSM algorithm, 248 patients had a normal CAP value, and 82 patients had an elevated CAP value in the study. Based on the CAP algorithm, the normal CAP group included 66 patients with no or mild fibrosis (LSM≤5.5 kPa), 126 patients with an uncertain diagnosis (5.5<LSM<9.5 kPa) and 56 patients with severe fibrosis or cirrhosis (LSM>9.5 kPa). The elevated CAP group included 29 patients with no or mild fibrosis (LSM<8 kPa), 37 patients with an uncertain diagnosis (8.0<LSM<14.8 kPa) and 16 patients with severe fibrosis or cirrhosis (LSM>14.8 kPa). We found that 79 (31.85%) patients had no significant fibrosis or a correct diagnosis of severe liver fibrosis/cirrhosis, 126 (50.81%) patients had an ambiguous diagnosis, and 43 (17.34%) patients had a misdiagnosis in the normal CAP group. In the elevated CAP group, 32 (39.02%) patients had no significant fibrosis or a correct diagnosis of severe liver fibrosis/cirrhosis, 37 (45.12%) patients had an ambiguous diagnosis, and 13 (15.85%) patients had a misdiagnosis in the elevated CAP group (Figure 4).

By comparing the diagnostic performance between the CAP-based LSM algorithm and the ALT-based LSM algorithm, we found that 112 (33.94%) patients had no significant fibrosis or a correct diagnosis of severe liver fibrosis/cirrhosis, 151 (45.76%) patients had an ambiguous diagnosis, and 67 (20.30%) patients had a misdiagnosis based on the ALT algorithm. However, based on the CAP algorithm, we found that 111 (33.64%) patients had no significant fibrosis or a correct diagnosis of severe liver fibrosis/cirrhosis, 163 (49.39%) patients had an ambiguous diagnosis, and 56 (16.97%) patients had a misdiagnosis. In this study, we found that the CAP-based LSM algorithm had a similar correct diagnosis rate and a lower misdiagnosis rate than the ALT-based LSM algorithm (Figure 4).

## DISCUSSION

In this study, 330 liver biopsy and transient elastography-proven CHB patients were enrolled. We found that CAP influenced the liver fibrosis stage, especially the lower fibrosis stages (S0-1). Importantly, the study showed that the LSM values were overestimated when assessing liver fibrosis in CHB patients with higher CAP values. The AUROC and diagnostic accuracy of LSM for liver fibrosis in the normal CAP group was higher than those in the evaluated CAP group. We therefore developed new liver stiffness cutoff values and an algorithm using LSM in the normal and elevated CAP groups. The new CAP-based algorithm has better diagnostic performance than the ALT-based algorithm.

Transient elastography has been widely applied routinely in clinical diagnosis because it is noninvasive and convenient to use. Patients with a higher fibrosis stage have higher LSM values. However, many factors other than liver fibrosis, such as inflammation and steatosis, may affect the LSM [16]. In most previous studies, steatosis was evaluated by semiquantitative scoring systems according to the percentage of affected hepatocytes by differing criteria [17, 18]. Those methods may produce a sampling error that could influence the results. Therefore, the CAP value, a new quantitative detection of hepatic steatosis, was used in the study. We found that CAP value is significantly correlated with LSM and liver fibrosis stage, especially the lower fibrosis stage (S0-1). Our findings are consistent with the report that CAP value is significantly correlated with a lower stage of fibrosis in NAFLD patients [19].

Because the CAP is significantly correlated with fibrosis stage, the study also evaluated the effect of CAP on LSM values at the same stages of liver fibrosis. We found that patients with higher CAP values had higher LSM values. This result demonstrated that the use of LSM could result in the overestimation of the liver fibrosis stage in some CHB patients with higher CAP values. To further examine the effect of CAP on LSM, the AUROCs, sensitivity, specificity, PPV, NPV and DA were further calculated in patients with different CAP values. The tests showed a higher DA for the CHB patients with normal CAP values than for the patients with elevated CAP values. Therefore, CAP also influenced the LSM diagnostic performance in CHB patients. These findings were consistent with the data from previously published reports [10, 20].

The TE results and optimal cutoff values varied from different cohorts and laboratories. Therefore, it is difficult to unify the optimal cutoff values [21]. In this study, we applied several optimal cutoff values of LSM in different stages of liver fibrosis in several CAP groups. We found that a higher fibrosis stage is associated with increased cutoff values. Several optimal cutoff values, with a higher sensitivity, specificity or Youden Index, may provide the highest rate of correct diagnoses in patients at various liver fibrosis stages. We found that higher LSM cutoff values were associated with higher CAP values.

We propose a diagnostic algorithm flowchart using LSM and CAP. The CAP value-based LSM diagnostic flowchart provides a similar accuracy rate regarding patients with no significant fibrosis or a correct diagnosis of severe liver fibrosis/cirrhosis and a lower rate of misdiagnosis than the ALT-based algorithm. The diagnostic flowchart using LSM and CAP values increases the DA for patients with severe liver fibrosis or cirrhosis compared to that using the LSM and ALT values. In addition, the CAP value-based diagnostic flowchart has several other advantages. First, our results showed that only BMI was independently significantly correlated with CAP, which was consistent with the results published in other studies [19, 22, 23]. This could suggest that CAP is less affected by other factors and thus could be a stable indicator of liver fibrosis. Second, both CAP and LSM are routine clinical markers tested by the same machine. The CAP value-based diagnostic flowchart is an economic and convenient addition to the clinical diagnostic process. Third, to the best of our knowledge, this is the first study to model the LSM cutoff values based on CAP. Importantly, the CAP-based LSM algorithm can be used in CHB patients who had large fluctuations during acute hepatitis, ALT flares and the use of liver-protecting drugs.

However, this study has some limitations. First, all patients were from a single institute, and this was a retrospective analysis. Larger multicenter studies and long-term clinical trials are necessary to confirm the findings of the present study. Second, this study enrolled only CHB patients with ALT<200 U/L. We need to include CHB patients with ALT≥200 U/L in a follow-up study.

In conclusion, the CAP value was found to be significantly correlated with LSM in CHB patients, especially in those at lower liver fibrosis stages. The diagnostic flowchart with the combination of CAP and LSM values is a more accurate, economic and convenient tool that can be used in the diagnosis of severe liver fibrosis or cirrhosis in CHB patients. Our findings provide a more flexible and accurate means of obtaining clinical diagnoses.

## MATERIALS AND METHODS

### Patients

CHB patients who underwent liver biopsy and transient elastography from January 2015 to August 2018 at Shengjing Hospital affiliated with China Medical University were enrolled in the study.

The enrolled CHB patients were positive for hepatitis B surface antigen (HBsAg) and HBV DNA for ≥6 months with persistently or intermittently abnormal ALT levels (> 40 IU/L). The exclusion criteria were as follows: (1) age less than 18 years; (2) other causes of liver disease; (3) other comorbid diseases (tumors, hematological diseases or human immunodeficiency virus infection); (4) significant alcohol consumption (> 20 g/d for women and > 30 g/d for men) for more than 5 years; (5) insufficient clinical data; and (6) serum ALT levels more than 5 times the upper limit of normal (UNL) (≥200 U/L).

### Ethics approval and consent

The study was approved by the Medical Ethics Committee of Shengjing Hospital of China Medical University (2018PS544K). All procedures in studies involving human participants were performed in accordance with the ethical standards of the institutional and/or national research committee and the 1975 Declaration of Helsinki and its later amendments or comparable ethical standards. Written informed consent was waived as this was a retrospective study using de-identified and aggregate data for analysis.

### Transient elastography

LSM and CAP were measured using transient elastography following the manufacturer’s instructions and were performed by trained and experienced operators. LSM value was defined as a valid measurement of at least 10 shots. LSM value was defined as unreliable with less than 10 valid shots, a success rate <60% and/or an interquartile range/median value (IQR/M) ≥30% of the measurement [24]. All enrolled patients underwent LSM within 6 months before liver biopsy. Hepatic steatosis was defined as more than 248 dB/m [25].

### Liver biopsy

Liver tissues were obtained by an experienced physician with strict adherence to the liver biopsy protocol. A liver sample was considered adequate if it was longer than 15 mm and contained six portal tracts or more. The specimens were fixed in formalin, processed for routine histopathological analysis, and reviewed by two experienced pathologists who were unaware of the patient’s clinical information.

The histopathological evaluation was performed by two trained and experienced pathologists who were blinded to the patient’s clinical data and transient elastography results. In case of disagreement, they reviewed the slides together to reach consensus. Liver fibrosis stage was classified from 0 to 4 according to Scheuer’s classification (for staging fibrosis: S0 = fibrosis was absent, S1 = portal fibrosis without septa, S2 = portal fibrosis with few septa, S3 = severe fibrosis, and S4 = cirrhosis; significant fibrosis was defined as stage S2-4, advanced fibrosis as S3-4, and cirrhosis as S4). Liver necroinflammation grade was classified from 0 to 4 according to Scheuer’s classification (for necroinflammation: G0 = no or minimal portal inflammation, G1 = portal inflammation, lobular inflammation without necrosis, G2 = mild piecemeal portal necrosis, focal lobular necrosis or acidophil bodies, G3 = moderate piece meal portal necrosis, severe local lobular cell damage, and G4 = severe piecemeal portal necrosis, damage includes bridging necrosis) [26].

### Clinical and laboratory data

Clinical data including age, sex, weight, height, dyslipidemia and alcohol consumption were recorded for all included patients. The day before liver biopsy, a 12-hour overnight fasting blood sample was collected to detect the levels of ALT, AST, ALP, GGT, triglycerides, TB, and total lipoprotein cholesterol. Serum HBsAg and hepatitis B e-antigen (HBeAg) were detected by microparticle enzyme immunoassay, and HBV DNA was measured using a TaqMan polymerase chain reaction assay.

### Statistical analysis

The results analysis was performed using SPSS, version 22.0 (SPSS Inc., Chicago, IL, United States). Data are reported as the medians (interquartile range, IQR) for continuous and nonnormally distributed variables and as frequencies or percentages for categorical variables. Spearman’s or Pearson’s correlation analysis was performed to analyze the relationship between CAP or LSM and clinical data. Data were compared between two groups of continuous variables using the Mann-Whitney *U* test. The diagnostic performance of transient elastography for the diagnosis of different stages of fibrosis was described by the AUROC, 95% confidence intervals (95%CIs), sensitivity (Sn), specificity (Sp), PPV, NPV and DA. A two-sided P < 0.05 was considered to indicate statistical significance.
